# Potential plant benefits of endophytic microorganisms associated with halophyte *Glycyrrhiza glabra* L.

**DOI:** 10.3934/microbiol.2024037

**Published:** 2024-09-30

**Authors:** Gulsanam Mardonova, Vyacheslav Shurigin, Farkhod Eshboev, Dilfuza Egamberdieva

**Affiliations:** 1 Faculty of Biology, National University of Uzbekistan, Tashkent 100174, Uzbekistan; 2 State Key Laboratory of Desert and Oasis Ecology, Xinjiang Key Laboratory of Biodiversity Conservation and Application in Arid Lands, Xinjiang Institute of Ecology and Geography, Chinese Academy of Sciences, Urumqi 830011, Xinjiang, China; 3 S. Yu. Yunusov Institute of the Chemistry of Plant Substances, Academy of Sciences of the Republic of Uzbekistan, Tashkent 100170, Uzbekistan; 4 School of Chemical Engineering, New Uzbekistan University, Movarounnahr Street 1, Mirzo Ulugbek District, Tashkent, 100000, Uzbekistan; 5 Institute of Fundamental and Applied Research, National Research University TIIAME, Tashkent 100000, Uzbekistan

**Keywords:** licorice, plant beneficial bacteria, antagonism, endophytes

## Abstract

In this study, bacteria associated with licorice (*Glycyrrhiza glabra* L.) were characterized through 16S rRNA gene analysis. Profiling of endophytic bacteria isolated from *Glycyrrhiza glabra* tissues revealed 18 isolates across the following genera: *Enterobacter* (4), *Pantoea* (3), *Bacillus* (2), *Paenibacillus* (2), *Achromobacter* (2), *Pseudomonas* (1), *Escherichia* (1), *Klebsiella* (1), *Citrobacter* (1), and *Kosakonia* (1). Furthermore, the beneficial features of bacterial isolates for plants were determined. The bacterial isolates showed the capacity to produce siderophores, hydrogen cyanide (HCN), indole-3-acetic acid (IAA), chitinase, protease, glucanase, lipase, and other enzymes. Seven bacterial isolates showed antagonistic activity against *F. culmorum*, *F. solani*, and *R. solani*. According to these results, licorice with antimicrobial properties may serve as a source for the selection of microorganisms that have antagonistic activity against plant fungal pathogens and may be considered potential candidates for the control of plant pathogens. The selected bacterial isolates, *P. polymyxa* GU1, *A. xylosoxidans* GU6, *P. azotoformans* GU7, and *P. agglomerans* GU18, increased root and shoot growth of licorice and were able to colonize the plant root. They can also serve as an active part of bioinoculants, improving plant growth.

## 1. Introduction

Endophytes are microorganisms that live inside plant cells, roots, stems, leaves, and tissues without causing any harmful effects on the plant. They are involved in plant growth and development processes by regulating plant metabolism [1]–[3]. Recent research provides strong evidence that these bacteria perform several beneficial functions for their host plants, such as promoting plant growth by aiding in the acquisition of nutrients (via nitrogen fixation, phosphate solubilization, or iron chelation), preventing pathogen infections through the production of antifungal or antibacterial metabolites, outcompeting pathogens for nutrients by producing siderophores, and enhancing systemic resistance in the plant [1],[4].

Bacteria that colonize root systems produce plant growth regulators, including auxins, cytokinins, and gibberellins. They also mobilize unavailable minerals, such as phosphorus and other essential elements, and inhibit the synthesis of ethylene through the activity of 1-aminocyclopropane-1-carboxylate (ACC) deaminase [4]–[6].

Glycyrrhiza, also known as licorice, is a legume with deep roots that can withstand salt, drought, and other environmental stresses. It is a popular plant for restoring salt-affected lands [7]. With roughly 20 species, this tall perennial shrub belongs to the leguminous Fabaceae (Leguminosae) family. It is primarily native to the Mediterranean region, Central Asia, and Southwestern Asia [8]. The chemical components of *G. glabra* include isoliquiritin, glycyrrhizin, glycyrrhetinic acid, and isoflavones. There have been reports of several pharmacological effects associated with licorice derivatives, including expectorant, anti-demulcent, anti-ulcer, anti-cancer, anti-inflammatory, and anti-diabetic properties [9]–[11]. Moreover, licorice is used as animal feed and in the phytoremediation of salt-affected soils. It is well adapted to salt-affected, arid lands and desert areas. Like many plants, licorice forms associations with various soil microorganisms, including nitrogen-fixing bacteria, which can enhance the plant's nutrient uptake [12]. In addition to nitrogen-fixing bacteria, other types of bacteria may also form associations with *Glycyrrhiza glabra*. These can include beneficial bacteria that promote plant growth, protect against pathogens, or assist in nutrient acquisition [13]. The specific bacterial communities associated with the plant can vary based on factors such as soil type, environmental conditions, and plant health.

Licorice can be susceptible to various pathogens, including fungi, bacteria, and viruses. For example, *Phytophthora* spp. cause root rot, and *Fusarium* spp. can cause wilt diseases that affect the vascular system, leading to plant growth inhibition [14]. Using environmentally friendly technologies to produce licorice is a significant strategy to ensure organic products. Employing plant-beneficial microorganisms is seen as an eco-friendly and alternative method of enhancing the fitness of medicinal plants [15]–[18]. Endophytic bacteria that live inside plants, including their roots, leaves, and stems, can be highly beneficial. Some mechanisms linked to these beneficial effects include the production of phytohormones, cell wall–degrading enzymes, hydrogen cyanide (HCN), and ACC-deaminase [19]–[22]. Numerous reports have documented the biological activity and diversity of endophytic bacteria associated with medicinal plants, such as *Ziziphora capitata* [23], *Aloe vera* [24], and *Origanum vulgare* [25]. Endophytes that colonize plant tissues are believed to play a major role in synthesizing physiologically active compounds and protecting plants from soil-transmitted diseases [26],[27]. Farhoui and coauthors [28] reported that sugar beets treated with bacterial isolates *Bacillus velezensis*, *Bacillus amyloliquefaciens*, and *Bacillus subtilis* exhibited a significant reduction in diseases caused by *Rhizoctonia solani* under greenhouse conditions. Genomic DNA extracted from each bacterial isolate revealed the presence of biosynthesis genes for lipopeptides such as iturin, surfactin, fengycin, and bacillomycin, which are known to exhibit strong antimicrobial activities. While there has been considerable research on the biological activity and phytochemical composition of licorice, studies on endophytes associated with licorice and their beneficial traits are relatively scarce. To enhance our understanding of the role of endophytes in plant growth and development, it is crucial to explore plant-microbe interactions and their physiological effects. The objectives of this study were to (1) identify culturable endophytic bacteria associated with licorice, (2) assess the plant beneficial traits of these bacterial isolates, and (3) determine the impact of bacterial inoculants on the tolerance of licorice plants to salt stress.

## 2. Materials and methods

### 2.1. Plant sample collection

Licorice (*Glycyrrhiza glabra* L.) was collected in June 2019 from Karakalpakstan, Uzbekistan, an area affected by salinity. The electrical conductivity of the saline soil was 7.8 dS/m. Using sterile gloves, ten separate plants, 12–15 meters apart, were collected, placed in zip-lock plastic bags, and transported to the lab for further analysis.

### 2.2. Isolation of endophytic bacteria

Plant roots and leaves were sterilized using NaClO (10%) and ethanol (70%), then rinsed in sterile water after 3 min. The roots and leaves (10 g) were ground with a sterile mortar and mixed with a phosphate buffer solution [29]. Bacteria were isolated from these mixtures using sterile phosphate-buffered saline and a nutrient-rich medium, tryptic soy agar (TSA) (BD, Difco Laboratories, USA), supplemented with 50 µg/mL of nystatin. After spreading 100 µL of the dilutions (10–10^5^) over TSA plates, the plates were incubated in a thermostat at 28 °C for 96 h. The sterility of the roots and leaves was verified by placing them on TSA plates [30].

### 2.3. Identification of bacteria

Bacteria were identified using 16S rRNA gene analysis. DNA isolation was performed by heat-treating bacterial cells according to Dashti et al. [31]. The presence of the isolated DNA was confirmed using horizontal gel electrophoresis. A portion of the 16S rRNA genes was amplified via polymerase chain reaction (PCR) using the following primers: 27F 5′-GAGTTTGATCCTGGCTCAG-3′ and 1492R 5′-GAAAGGAGGTGATCCAGCC-3′ (both from Sigma-Aldrich, St. Louis, Missouri, USA) [32]. The amplified 16S rRNA gene fragments were examined for restriction fragment length polymorphism, and bacteria with the same genotype were eliminated according to Jinneman et al. [33]. The sequencing of PCR products was performed using the ABI PRISM BigDye 3.1 Terminator Cycle Sequencing Ready Reaction Kit (Applied Biosystems). The nucleotide sequences of the 16S rRNA gene were aligned using EMBOSS Explorer (http://emboss.bioinformatics.nl/) and Chromas (v.2.6.5) software. The sequences of the isolates' 16S rRNA genes were compared with those in GenBank (http://www.ncbi.nlm.nih.gov/) using the Basic Local Alignment Search Tool (BLAST) for identification. A FASTA file containing the 16S rRNA sequences of the isolates and related strains from GenBank, obtained after multiple alignments with Clustal Omega (https://www.ebi.ac.uk/Tools/msa/clustalo/), was used for constructing a phylogenetic tree. The percentage of replicate trees in which the associated taxa clustered together in the bootstrap test (500 replicates) is shown next to the branches. The tree is drawn to scale, with branch lengths in the same units as the evolutionary distances used to infer the phylogenetic tree. These evolutionary distances were computed using the maximum composite likelihood method [34] and are expressed in units of the number of base substitutions per site. This analysis involved 36 nucleotide sequences, with all ambiguous positions removed for each sequence pair (pairwise deletion option). The final dataset comprised 1,567 positions. Evolutionary analyses were conducted using MEGA X [35].

The nucleotide sequences of the 16S rRNA gene were registered in GenBank and received the accession numbers OQ874308 to OQ874325.

### 2.4. Beneficial traits of plant endophytes

The capability of bacterial isolates to synthesize hydrogen cyanide (HCN) was investigated using TSA media. A sterilized filter paper saturated with a 1% solution of picric acid and 2% sodium carbonate was placed in the upper lid of a Petri dish. The Petri dish was sealed with parafilm and incubated at 28 °C for 3 days. A change in the paper color from yellow to dark blue indicated HCN production [36]. The method by Schwyn and Neilands [37] was used to determine the bacterial isolates' capacity to produce siderophores. Briefly, the bacterial isolates were plated on standard blue agar with chrome azurol sulfonate (CAS) and incubated at 28 °C. After 5 days, a pink-orange zone around the bacterial colonies indicated siderophore production. Protease activity was detected by plating bacterial isolates on TSA amended with 5% skimmed milk. After 4 days of incubation at 28 °C, the appearance of a halo around the colonies indicated the presence of extracellular protease [38].

The synthesis of β-1,3 glucanase was determined using the method described by Walsh et al. [39]. Bacterial isolates were plated on nutrient agar amended with the glucan substrate lichenan, and after 4 days of incubation, a clear zone around the colonies indicated substrate degradation. Cellulase activity was detected using the substrate carboxymethylcellulose in top-agar plates, following the method by Malleswari and Bagyanarayana [40]. The lipase activity of bacterial isolates was assessed using the Tween lipase indicator test [41]. Briefly, bacterial isolates were grown on LC agar (LB agar containing 10 mM MgSO_4_ and 5 mM CaCl_2_) with 2% Tween 80 at 28 °C. After 5 days, the degradation of Tween was indicated by a clear halo around the bacterial inoculum. Using the technique outlined by Bano and Musarrat [42], the synthesis of IAA (indole-3-acetic acid) by endophytic isolates was investigated. The bacterial isolates were grown in LC medium with tryptophan (500 µg/mL) and incubated at 28 °C. One milliliter of supernatant was transferred to a fresh tube, to which 100 µL of 10 mM orthophosphoric acid and 2 mL of reagent (1 mL of 0.5 M FeCl_3_ in 50 mL of 35% HClO_4_) were added. After 25 min, the absorbance of the developed pink color was measured at 530 nm.

ACC-deaminase synthesis was investigated using 1-aminocyclopropane-1-carboxylic acid (ACC) as the sole nitrogen source [43]. The ability of endophytic bacterial isolates to inhibit plant pathogenic fungi *Fusarium oxysporum*, *Fusarium culmorum*, and *Rhizoctonia solani* was evaluated following the method described by Egamberdieva et al. [44]. Fungal strains were grown on agar plates at 28 °C for 5 days. Disks (5 mm in diameter) of fresh fungal cultures were cut out and placed in the center of a 9 cm Petri dish. Bacteria (grown on peptone agar plates) were streaked perpendicular to the fungi on the test plates. The plates were incubated at 30 °C for 7 days until the fungi had grown over control plates without bacteria. Anti-fungal activity was recorded as the width of the zone of growth inhibition between the fungus and the test bacterium.

### 2.5. Germination of seeds

*G. glabra* seeds were surface-sterilized by immersing them for 5 min in a 70% sulfuric acid solution, followed by five rinses with sterile distilled water, and then a 3 min immersion in 70% ethanol. The bacterial strains were grown overnight in nutrient medium. One milliliter of the overnight culture was centrifuged at 13,000 × g, and the supernatant was discarded. The cells were washed with 1 mL phosphate-buffered saline (PBS) and re-suspended in PBS. The cell suspension was adjusted to an OD_620_ of 0.1, corresponding to a cell density of approximately 10^8^ cells/mL. Inoculation was performed by immersing the seeds in the bacterial suspension.

Germination tests were conducted using Petri dishes (Ø 85 mm × 15 mm) filled with 1% water agar supplemented with 50 mM NaCl. Twenty surface-sterilized licorice seeds were placed on each Petri dish, with three replications. To prevent moisture evaporation, the Petri dishes were covered with a polyethylene sheet and maintained at 28 °C in a plant growth chamber. During the six-day period, seeds were monitored, and the percentage of germination was recorded. Seeds were considered to have germinated when radicles emerged and reached a length of more than 0.5 cm. Ten days after sowing, the lengths of the seedlings were measured and recorded.

### 2.6. Plant growth in a gnotobiotic sand system

The effect of bacterial isolates on the growth of licorice seedlings exposed to 50 mM NaCl stress was examined using six replicates in gnotobiotic sand tubes (25 mm in diameter by 200 mm in length), as described by Simons et al. [45]. Sixty grams of a sterilized mixture of washed sand and vermiculite (1:1) were soaked in 6 mL of diluted nitrogen-free Jensen nutrient solution supplemented with 50 mM NaCl. Surface-sterilized licorice seeds were allowed to germinate on 1% water agar for three days at 28 °C in the dark. One germinated seed per sterile glass tube was planted after being submerged in a bacterial suspension (10^8^ CFU/mL) for 15 min. The seedlings were grown in a growth cabinet under a light regime of 16 h light at 22 °C and 8 h darkness at 16 °C. After 14 days, the lengths of the roots and shoots, as well as the fresh weight of the entire plant, were measured.

### 2.7. Colonization of root tips by bacteria

Using the previously described gnotobiotic sand tubes, the colonization of licorice root tips by bacterial isolates was examined. The methods for cultivating and preparing bacterial inoculants and inoculating seeds, as described earlier, were employed. After two weeks, the seedlings were removed from the sand, and 1 cm of the root tips were excised and placed into a tube with 1 mL of PBS. The root tips were vortexed in PBS to dislodge any bacterial cells. Following a series of dilutions, homogenates were spread on agar plates at 10^3^ and 10^4^ dilutions. Bacterial colonies on TSA were counted after three days of incubation at 28 °C. The colony-forming units (CFU) per 1 cm of root tip were used to calculate the quantity of bacterial cells.

### 2.8. Plant growth in pots

Bacterial isolates were grown for 72 h in tryptic soy broth (TSB, Sigma-Aldrich), and their suspensions were adjusted to an optical density of 0.1 (OD_620_ = 0.1) at 620 nm, corresponding to approximately 10^8^ cells/mL. Licorice seeds were immersed for 10 min in a bacterial suspension with a concentration of 10^7^ colony-forming units (CFU) per milliliter. Plastic pots, 12 cm in diameter and 16 cm in depth, were filled with 500 g of soil collected from salt-affected land in the Sirdarya province of Uzbekistan. One seed per pot was sown. The experiment included two treatments—seeds that were not treated with bacteria and seeds that were inoculated with bacteria. Each treatment was replicated three times in a completely randomized block design. The plants were grown with day temperatures of 24–28 °C and night temperatures of 14–16 °C. After eight weeks, the lengths of the shoots and roots, as well as their dry weights, were assessed.

### 2.9. Statistical analyses

The analysis of variance tool in Microsoft Excel 2010 was used to determine the statistical significance of the data. Student's t-test was used to perform comparisons. The least significant difference (LSD) test (P = 0.05) was used to compare means.

## 3. Results

### 3.1. Isolation and identification of cultivable endophytic bacteria

**Figure 1.**
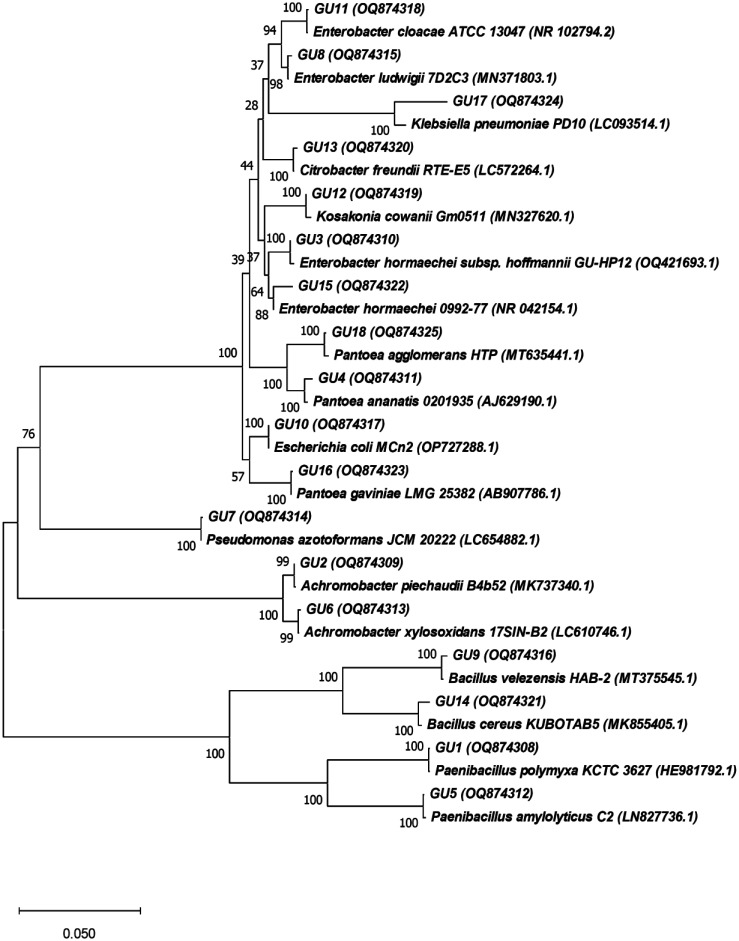
Phylogenetic tree of endophytic bacteria (GU1–GU18) from *Glycyrrhiza glabra* with the closest relatives registered in GenBank of NCBI.

A total of 55 bacterial strains were isolated from the plant tissues of *G. glabra*. After RFLP analysis, only 18 strains remained. These strains were identified using the BLAST (Basic Local Alignment Search Tool) and matched with corresponding strains from the NCBI GenBank. The strains were found to be 99.79%–100% identical to their closest relatives registered in GenBank®. Sequence similarities of the endophytic bacteria isolated from *G. glabra* are presented in Table 1. The lengths of the identified nucleotide sequences of the 16S rRNA genes of the isolates ranged from 1354 to 1492 bp, which is considered adequate for reliable identification based on 16S rRNA gene analysis using the BLAST tool. All isolated strains were assigned accession numbers, as shown in Table 1 and Figure 1. As shown in Table 1, the tissues of *G. glabra* harbored 18 species belonging to two phyla: *Pseudomonadota* (GU2, GU3, GU4, GU6, GU7, GU8, GU10, GU11, GU12, GU13, GU15, GU16, GU17, and GU18) and *Bacillota* (GU1, GU5, GU9, and GU14). The phylum *Pseudomonadota* comprised 2 classes: *Gammaproteobacteria* (GU3, GU4, GU7, GU8, GU10, GU11, GU12, GU13, GU15, GU16, GU17, and GU18) and *Betaproteobacteria* (GU2 and GU6). The phylum *Bacillota* was presented by a single class *Bacilli* with the strains given above.

Profiling of endophytic bacteria isolated from the tissues of *G. glabra* demonstrated that these included 18 isolates belonging to the genera *Enterobacter* (4), *Pantoea* (3), *Bacillus* (2), *Paenibacillus* (2), *Achromobacter* (2), *Pseudomonas* (1), *Escherichia* (1), *Klebsiella* (1), *Citrobacter* (1), and *Kosakonia* (1) (Figure 1).

**Table 1.** Endophytic bacteria isolated from *Glycyrrhiza glabra* and their closest relatives from GenBank based on 16S rRNA gene resemblance.

Isolated strains deposited to GenBank				Closest match in GenBank		
Strain	Species	Query length (bp)	Accession number	Reference strain	Accession number	Identity (%)
GU1	*Paenibacillus polymyxa*	1465	OQ874308	*Paenibacillus polymyxa* KCTC 3627	HE981792.1	99.93
GU2	*Achromobacter piechaudii*	1375	OQ874309	*Achromobacter piechaudii* B4b52	MK737340.1	99.93
GU3	*Enterobacter hormaechei*	1439	OQ874310	*Enterobacter hormaechei* subsp. Hoffmannii GU-HP12	OQ421693.1	99.86
GU4	*Pantoea ananatis*	1464	OQ874311	*Pantoea ananatis* 0201935	AJ629190.1	99.8
GU5	*Paenibacillus amylolyticus*	1419	OQ874312	*Paenibacillus amylolyticus* C2	LN827736.1	99.93
GU6	*Achromobacter xylosoxidans*	1423	OQ874313	*Achromobacter xylosoxidans* 17SIN-B2	LC610746.1	99.86
GU7	*Pseudomonas azotoformans*	1456	OQ874314	*Pseudomonas azotoformans* JCM 20222	LC654882.1	99.93
GU8	*Enterobacter ludwigii*	1461	OQ874315	*Enterobacter ludwigii* 7D2C3	MN371803.1	99.79
GU9	*Bacillus velezensis*	1449	OQ874316	*Bacillus velezensis* HAB-2	MT375545.1	99.93
GU10	*Escherichia coli*	1457	OQ874317	*Escherichia coli* MCn2	OP727288.1	100
GU11	*Enterobacter cloacae*	1464	OQ874318	*Enterobacter cloacae* ATCC 13047	NR_102794.2	100
GU12	*Kosakonia cowanii*	1404	OQ874319	*Kosakonia cowanii* Gm0511	MN327620.1	99.86
GU13	*Citrobacter freundii*	1465	OQ874320	*Citrobacter freundii* RTE-E5	LC572264.1	99.8
GU14	*Bacillus cereus*	1492	OQ874321	*Bacillus cereus* KUBOTAB5	MK855405.1	99.87
GU15	*Enterobacter hormaechei*	1354	OQ874322	*Enterobacter hormaechei* 0992-77	NR_042154.1	99.85
GU16	*Pantoea gaviniae*	1483	OQ874323	*Pantoea gaviniae* LMG 25382	AB907786.1	99.87
GU17	*Klebsiella pneumoniae*	1431	OQ874324	*Klebsiella pneumoniae* PD10	LC093514.1	99.79
GU18	*Pantoea agglomerans*	1422	OQ874325	*Pantoea agglomerans* HTP	MT635441.1	99.79

### 3.2. Plant-beneficial traits

**Table 2.** Beneficial traits of endophytic bacteria associated with *Glycyrrhiza glabra*.

Bacterial endophytes	HCN	Lipase	Glucanase	Chitinase	IAA	ACC-deaminase	Siderophore	*F. oxysporum*	*F. culmorum*	*R. solani*
*P. polymyxa* GU1	+	-	+	+	+	+	–	+	+	+
*A. piechaudii* GU2	-	-	-	-	–	+	–	–	–	–
*E. hormaechei* GU3	+	-	-	+	–	–	+	–	–	–
*P. ananatis* GU4	-	+	-	+	–	–	–	–	–	–
*P. amylolyticus* GU5	+	-	+	+	+	+	–	–	+	+
*A. xylosoxidans* GU6	+	-	+	+	+	–	+	–	–	–
*P. azotoformans* GU7	+	+	-	+	+	–	–	+	+	+
*E. ludwigii* GU8	+	+	-	-	–	+	–	–	–	–
*B. velezensis* GU9	+	+	+	-	–	–	–	–	–	–
*E. coli* GU10	-	-	+	-	–	–	–	–	–	–
*E. cloacae* GU11	+	+	-	-	–	+	–	–	–	–
*K. cowanii* GU12	-	+	-	+	–	–	–	–	–	–
*C. freundii* GU13	+	-	+	-	–	–	–	–	–	–
*B. cereus* GU14	+	+	+	-	+	+	+	–	–	–
*E. hormaechei* GU15	-	+	+	-	–	+	+	+	–	+
*P. gaviniae* GU16	-	-	-	-	–	–	–	–	–	–
*K. pneumoniae* GU17	-	+	-	+	–	–	–	–	–	–
*P. agglomerans* GU18	-	-	+	+	+	+	–	+	+	–

“+” positive to the tested activity.

Table 2 provides findings about the plant-beneficial characteristics of endophytic bacteria. Bacterial isolates *P. polymyxa* GU1, *P. amylolyticus* GU5, *B. cereus* GU14, *E. hormaechei* GU15, and *P. agglomerans* GU18 produced IAA. Siderophore production was observed in 4 out of 18 bacterial isolates. The eight isolates *P. polymyxa* GU1, *A. piechaudii* GU2, *P. amylolyticus* GU5, *E. ludwigii* GU8, *E. cloacae* GU11, *B. cereus* GU14, *E. hormaechei* GU15, and *P. agglomerans* GU18 showed ACC deaminase production and nine of the strains showed hydrogen cyanide (HCN) production. The strains were also tested for fungal cell wall–degrading enzymes (protease, cellulase, and lipase) production. It was revealed that 11 bacterial isolates produced at least two tested enzymes (Table 2). The antifungal activity of endophytic bacterial isolates was evaluated against three plant pathogenic fungi, *F. culmorum*, *F. oxysporum*, and *R. solani* (Table 2). Among all tested endophytic bacteria, the five isolates *P. polymyxa* GU1, *P. amylolyticus* GU5, *P. azotoformans* GU7, *E. hormaechei* GU15, and *P. agglomerans* GU18 exhibited strong inhibition against three tested plant pathogenic fungi, namely *F. culmorum, F. solani*, and *R. solani*.

### 3.3. Seed germination

**Figure 2.**
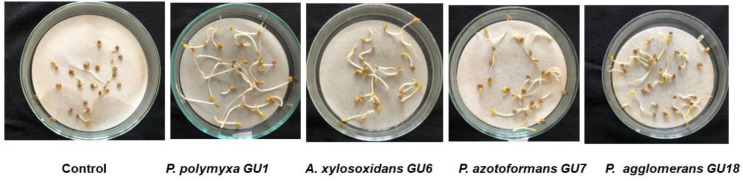
The effect of bacterial isolates on seed germination.

We also examined the effect of bacterial inoculants on the seed germination of *Glycyrrhiza glabra*. The results revealed that the germination rate of non-inoculated *G. glabra* seeds was 57% ± 2.1%, which was lower compared to seeds inoculated with bacteria. Inoculation with bacterial isolates improved seed germination. Specifically, *P. polymyxa* GU1, *E. hormaechei* GU15, and *P. agglomerans* GU18 increased germination rates to 75% ± 2.4%, 70% ± 3.1%, and 70% ± 2.9%, respectively. In contrast, *P. amylolyticus* GU5, *P. azotoformans* GU7, and *B. cereus* GU14 exhibited lower germination rates, at 65% ± 3.2% (Figure 2).

The bacterial isolates also stimulated seedling growth compared to control seeds. After 10 days incubation, the seedling length of control was 2.45 cm, whereas *P. polymyxa* GU1, *A. xylosoxidans* GU6, and *P. azotoformans* GU7 increased seedling length by 4.25 cm. *P. amyloliticus* GU5 and *E. hormaechei* GU15 had no effect on seedling growth and development (Table 3).

**Table 3.** The effect of bacterial inoculation on seed germination and seedling growth of *Glycyrrhiza glabra*.

Bacterial isolates	Seed germination (%)	Seedling length (cm)
Control (no bacterial inoculation)	57 ± 2.1	2.54 ± 0.7
*Paenibacillus polymyxa* GU1	75 ± 2.4*	4.75 ± 0.9*
*Paenibacillus amylolyticus* GU5	65 ± 2.9	2.6 ± 0.3
*Achromobacter xylosoxidans* GU6	68 ± 2.2*	4.25 ± 0.4*
*Pseudomonas azotoformans* GU7	65 ± 1.9	4.75 ± 0.9*
*Bacillus cereus* GU14	65 ± 2.0	3.65 ± 0.7
*Enterobacter hormaechei* GU15	70 ± 3.1*	2.95 ± 0.3
*Pantoea agglomerans* GU18	70 ± 2.9*	3.35 ± 0.6*

*Note: Asterisks indicate the level of statistical significance: p ≤ 0.05.

### 3.4. Response of salt-stressed licorice in a gnotobiotic sand system to bacterial inoculation

**Figure 3.**
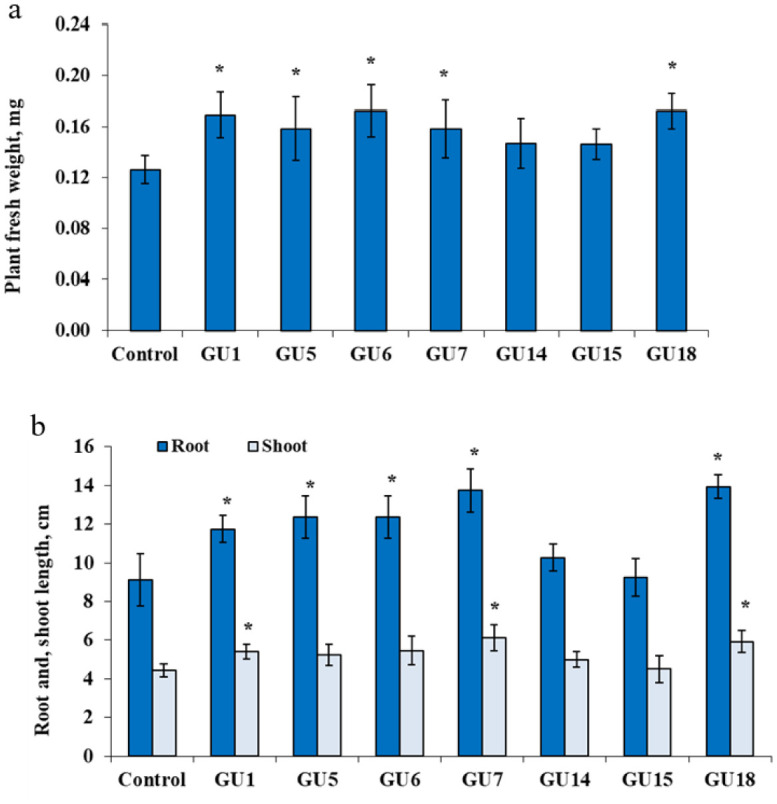
Effect of inoculation with the bacterial isolates (*P. polymyxa* GU1, *P. amylolyticus* GU5, *A. xylosoxidans* GU6, *P. azotoformans* GU7, *B. cereus* GU14, *E. hormaechei* GU15 and *P. agglomerans* GU18) on the fresh weight of whole plants (a) and on shoots and roots length (b) of salt-stressed *G. glabra* seedlings. Columns represent means for six seedlings (N = 6) with error bars showing standard error.

The initial salt tolerance of *G. glabra* and the response of plants to salt stress (50 mM NaCl) following endophytic bacterial inoculation were evaluated. Our results demonstrated that *P. polymyxa* GU1, *P. amylolyticus* GU5, *A. xylosoxidans* GU6, *P. azotoformans* GU7, and *P. agglomerans* GU18 improved the fresh weight, root length, and shoot length of licorice. Specifically, fresh weight increased by 36%, root length by 52%, and shoot length by 39% compared with the uninoculated control. However, there was no significant difference compared to plants inoculated with *B. cereus* GU14 and *E. hormaechei* GU15 (Figure 3a,b). The bacterial isolates *P. agglomerans* GU18 and *P. azotoformans* GU7 exhibited the most pronounced stimulating effects.

### 3.5. Root colonization by endophytic bacteria

**Figure 4.**
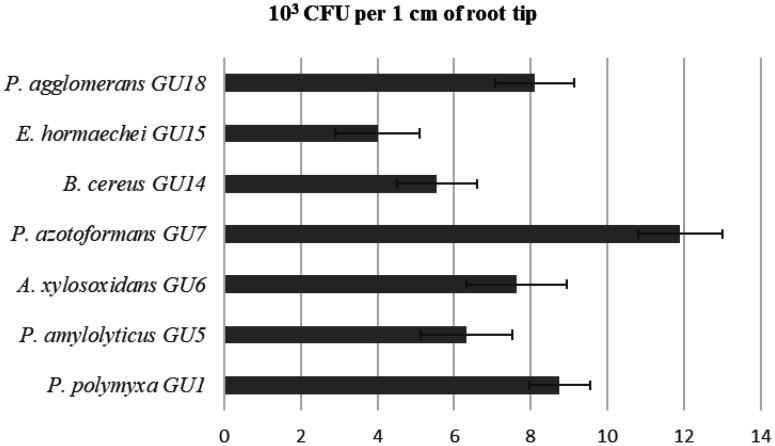
Colonization ability of selected endophytic bacteria in plant root (CFU cm root), (*P. polymyxa* GU1, *P. amylolyticus* GU5, *A. xylosoxidans* GU6, *P. azotoformans* GU7, *B. cereus* GU14, *E. hormaechei* GU15, *P. agglomerans* GU18).

We have also determined the colonization of introduced bacteria in the root of licorice. Our experiment showed that CFU counts of *P. azotoformans* GU7 were 11.9 × 10^3^ CFU/cm of root tip; for isolates *P. polymyxa* GU1 and *P. agglomerans* GU18, it was 8.76 and 8.10 × 10^3^ CFU/cm of root tip, respectively. Lower colonization was observed by *B. cereus* GU14 and *E. hormaechei* GU15, being 5.55 and 4.01 × 10^3^ CFU/cm of root tip (Figure 4).

### 3.6. Plant growth and nutrient uptake of licorice inoculated with bacterial inoculants

**Figure 5.**
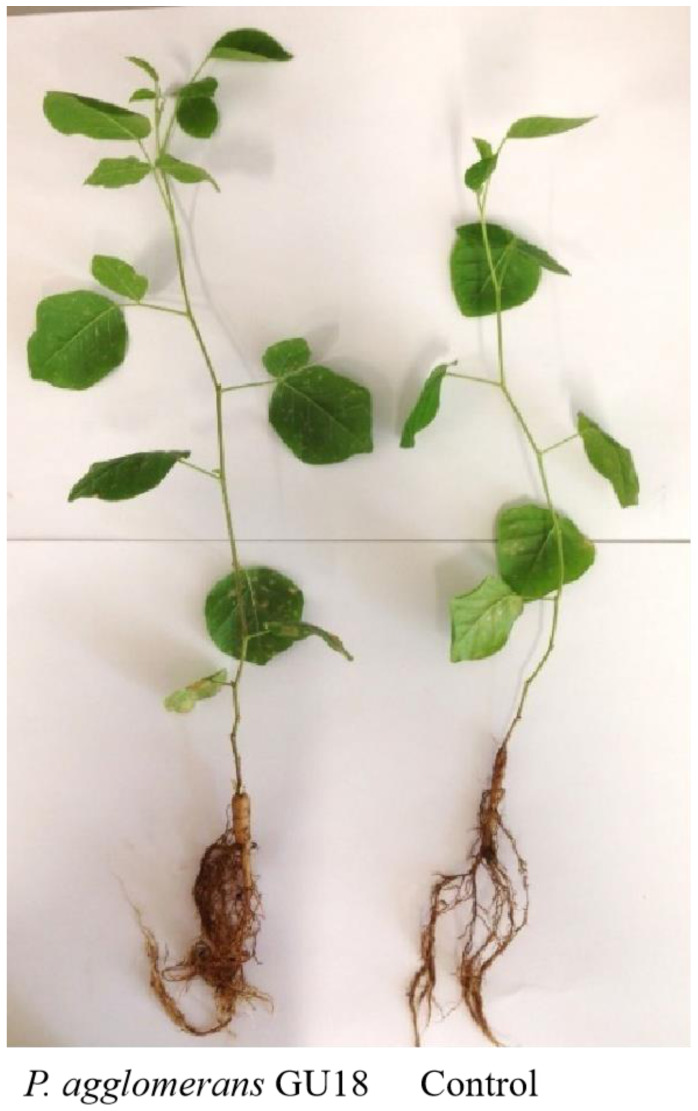
Phenotype of *G. glabra* plants uninoculated and inoculated with *P. agglomerans* GU18 and grown in saline soil for eight weeks.

The effect of bacterial isolates selected from previous experiments on plant growth in pots with saline soil was further investigated under greenhouse conditions. Plants were grown for 8 weeks in saline soil. The results obtained from this pot experiment were similar to those obtained from the short-term gnotobiotic experiment. After bacterial inoculation with *P. polymyxa* GU1, *A. xylosoxidans* GU6, *P. azotoformans* GU7, and *P. agglomerans* GU18, the shoot fresh weights were increased by 69%, 42%, 57%, and 55%, and roots were increased by 83%, 26%, 61%, and 66%, respectively (Table 4). In comparison with the uninoculated plant, the co-inoculation of bacterial isolates increased shoot and root dry weights by 76% (Table 4). *P. polymyxa* GU1, *A. xylosoxidans* GU6, *P. azotoformans* GU7, and *P. agglomerans* GU18 performed the best and, in comparison with uninoculated plants, the shoot and root weights increased by 71%, 51%, 74%, and 76%, respectively (Table 4, Figure 5).

**Table 4.** Effect of bacterial inoculation on shoot and root growth of *Glycyrrhiza glabra*.

Plant	Control	GU1	GU5	GU6	GU7	GU14	GU15	GU18
Fresh weight	
Shoot	6.38 ± 0.47	10.83 ± 0.55*	6.25 ± 0.39	9.07 ± 0.39*	10.03 ± 0.33*	7.66 ± 0.45	7.36 ± 0.50*	9.05 ± 0.43*
Root	2.90 ± 0.24	5.60 ± 0.41*	2.80 ± 23	3.66 ± 0.39*	4.69 ± 0.25*	3.1 ± 0.23	4.01 ± 0.38*	4.83 ± 0.40*
Dry weight	
Shoot	1.98 ± 0.11	3.21 ± 0.38*	1.84 ± 0.12	2.93 ± 0.15*	3.17 ± 0.35*	2.31 ± 0.11	2.09 ± 0.09	3.41 ± 0.17*
Root	1.12 ± 0.19	1.91 ± 0.16*	1.06 ± 0.15	1.66 ± 0.12*	1.95 ± 0.22*	1.39 ± 0.10	1.35 ± 0.18	1.90 ± 0.12*

*Note: Asterisks indicate the level of statistical significance: p ≤ 0.05, (*P. polymyxa* GU1, *P. amylolyticus* GU5, *A. xylosoxidans* GU6, *P. azotoformans* GU7, *B. cereus* GU14, *E. hormaechei* GU15, *P. agglomerans* GU18).

## 4. Discussion

To the best of our knowledge, this is the first study to examine endophytic bacteria associated with *G. glabra* growing in salt-affected land in Uzbekistan. Profiling of endophytic bacteria isolated from the tissues of *Glycyrrhiza glabra* revealed 18 isolates belonging to the following genera: *Enterobacter* (4), *Pantoea* (3), *Bacillus* (2), *Paenibacillus* (2), *Achromobacter* (2), *Pseudomonas* (1), *Escherichia* (1), *Klebsiella* (1), *Citrobacter* (1), and *Kosakonia* (1). Similar bacterial species have been reported in other medicinal plants: *Bacillus cereus* from *Dicoma anomala* [46], *Paenibacillus polymyxa* SK1 from *Lilium lancifolium* [47], *Pseudomonas amylolyticus* from *Coix lachryma-jobi* [48], and *Pantoea ananatis* from *Iris pseudacorus* [49]. We have also observed that several bacterial isolates belonging to *A. piechaudii*, *E. hormaeche*, *A. xylosoxidans*, *E. ludwigii*, *E. coli*, *E. cloacae*, *K. cowanii*, *C. freundii*, *B. cereus*, *E. hormaechei*, and *K. pneumoniae* are potential human pathogens. While many bacteria associated with plants are beneficial, some may pose a threat to human health [50]. Saline environments can affect the composition and diversity of microbial communities, potentially impacting the abundance of pathogenic species. Saline soils may support the growth of certain human pathogenic bacteria, leading to human diseases through direct contact with contaminated plants or indirect exposure via contaminated food or water [51].

Furthermore, our investigation revealed that several bacterial isolates exhibited plant-beneficial traits. Previous studies have reported that bacterial isolates stimulate plant growth, enhance nutrient acquisition, and increase plant stress tolerance to abiotic stresses such as drought and salinity by synthesizing biologically active substances [52]–[54]. We have observed antagonistic activity of several bacterial isolates against the plant pathogenic fungi *F. oxysporum, F. solani*, and *R. solani*. These bacteria may protect plants from pathogenic fungi by producing antibiotics or competing for nutrients and niches [55],[56]. Previous studies on plant-associated bacteria from *Hypericum perforatum* and *Chelidonium majus* L. revealed higher percentages of endophytes with antifungal characteristics [57]. Evidence suggests that the physiological processes of endophytic bacteria residing within plant tissues may be influenced by the biologically active components of medicinal plants. These endophytic bacteria can mimic the biological activity and metabolite synthesis of their host plants. For example, bacteria isolated from the medicinal plants *Matricaria chamomilla* and *Calendula officinalis* demonstrated antifungal activities comparable to those of the plant extracts [57]. In another study, bacteria associated with *Aloe vera* exhibited antibacterial activity against human pathogenic bacteria, such as *S. aureus*, *Streptococcus pyogenes*, *P. aeruginosa*, and *E. coli*, and produced bioactive compounds with antimicrobial activities [58]. Furthermore, studies have shown that the antagonistic properties of endophytic bacteria can efficiently reduce fungal diseases without harming the host [59].

Numerous basic mechanisms underlying the beneficial effects of endophytic bacteria have been documented in previous reports [60]. These include the synthesis of phytohormones, hydrogen cyanide (HCN), siderophores, ACC-deaminase, enzymes that degrade fungal cell walls, and phosphate solubilization. In our study, six bacterial strains produced the phytohormone auxin, ten strains synthesized hydrogen cyanide (HCN), and nine bacterial isolates produced at least two of the three tested fungal cell wall–degrading enzymes: chitinase, glucanase, and lipase. It is known that one of the primary mechanisms for suppressing plant pathogens involves bacterial production of chitinase, which degrades fungal cell walls, lipase, which breaks down certain lipids associated with fungal cell walls, and β-1,3-glucanase, which degrades cell wall carbohydrates. It has also been reported that bacteria producing hydrogen cyanide (HCN) can inhibit the growth of fungal pathogens [61]. Many studies have documented the production of phytohormones by bacterial strains associated with plants. Phytohormone-producing bacteria stimulate root and shoot growth, enhance nutrient acquisition, and improve the yield of various crop and medicinal plants [62],[63]. For instance, indole-3-acetic acid (IAA) promotes root elongation, enhances root hair formation, and facilitates better nutrient and water uptake. This enhances plant anchorage and stability, which is crucial for plants growing in stressful environments like saline soils. In our study, eight out of eighteen endophytic bacterial isolates were able to produce ACC deaminase (1-aminocyclopropane-1-carboxylate deaminase). Ethylene, a plant hormone involved in various physiological functions including stress responses, is derived from ACC. Bacteria that produce ACC deaminase can lower plant ethylene levels by breaking down ACC, thereby reducing the negative effects of ethylene on plant growth and development under stress conditions [64].

Seven bacterial isolates improved seed germination and seedling growth and were further tested in pot experiments. Four bacterial isolates, *P. polymyxa* GU1, *A. xylosoxidans* GU6, *P. azotoformans* GU7, and *P. agglomerans* GU18, significantly increased root and shoot of licorice in saline soil. There were many reports on the positive effect of endophytic bacteria on plant growth of medicinal plants [65]–[67]. According to Sudarshna and Sharma [68], endophytic bacteria isolated from the *Trillium govanianum* with IAA-, siderophore-, and ACC deaminase-producing ability enhanced plant growth and nutrient uptake from soil. In another study, *Pelargonium graveolens–*associated bacteria with various plant-beneficial traits increased plant dry weight and essential oils concentration [69]. This beneficial effect largely stems from the bacteria's ability to colonize the plant's root system, which is essential for fostering positive interactions between the bacteria and the plant. Bacteria employ various mechanisms to facilitate root colonization, including chemotaxis toward root exudates and biofilm formation on root surfaces. In our study, five bacterial isolates that demonstrated the greatest potential for stimulating plant growth were also able to successfully colonize the roots of licorice.

## 5. Conclusions

In this study, we identified endophytic bacteria associated with *Glycyrrhiza glabra* from a salt-affected region of Uzbekistan. These bacteria belong to the genera *Enterobacter*, *Pantoea*, *Bacillus*, *Paenibacillus*, *Achromobacter*, *Pseudomonas*, *Escherichia*, *Klebsiella*, *Citrobacter*, and *Kosakonia*. The bacterial isolates demonstrated the ability to produce siderophores, hydrogen cyanide (HCN), indole-3-acetic acid (IAA), and various enzymes, and exhibited antagonistic activity against *F. culmorum*, *F. solani*, and *R. solani*. These isolates not only enhanced root and shoot growth in licorice but also successfully colonized the rhizosphere. Our findings underscore the potential of these specific bacterial strains as effective microbial inoculants to boost licorice growth in saline soils. Using these inoculants could greatly boost agricultural productivity in saline conditions, leading to better licorice cultivation and potential economic advantages. Further research and field trials are needed to optimize inoculant formulations and confirm their effectiveness across varying environmental conditions.

## Use of AI tools declaration

The authors declare they have not used Artificial Intelligence (AI) tools in the creation of this article.

## References

[1] Santos MLD, Berlitz DL, Wiest SLF (2018). Benefits associated with the interaction of endophytic bacteria and plants. Braz Arch Biol Technol.

[2] Egamberdieva D, Alimov J, Shurigin V (2022). Diversity and plant growth-promoting ability of endophytic, halotolerant bacteria associated with *Tetragonia tetragonioides* (Pall.) Kuntze. Plants.

[3] Egamberdieva D, Ma H, Reckling M (2022). Interactive effects of biochar and N and P nutrients on the symbiotic performance, growth, and nutrient uptake of soybean (*Glycine max* L.). Agronomy.

[4] Weyens N, van der Lelie D, Taghavi S (2009). Phytoremediation: Plant–endophyte partnerships take the challenge. Curr Opin Biotechnol.

[5] Shurigin V, Alaylar B, Davranov K (2021). Diversity and biological activity of culturable endophytic bacteria associated with marigold (*Calendula officinalis* L.). AIMS Microbiol.

[6] Shurigin V, Egamberdieva D, Li L (2020). Endophytic bacteria associated with halophyte *Seidlitzia rosmarinus* Ehrenb. ex Boiss. from saline soil of Uzbekistan and their plant beneficial traits. J Arid Land.

[7] Marui A, Nagafuchi T, Shinogi Y (2012). Soil physical properties to grow the wild licorice at semi-arid area in Mongolia. J Arid Land Studies.

[8] Lewis G, Schrire B, Mackinder B (2005). Legumes of the world.

[9] Hayashi H, Hattori S, Inoue K (2003). Field survey of *Glycyrrhiza* plants in Central Asia (1). Characterization of *G. uralensis*, *G. glabra* and the putative intermediate collected in Kazakhstan. Biol Pharm Bull.

[10] Patil SM, Patil MB, Sapkale GN (2009). Antimicrobial activity of *Glycyrrhiza glabra* Linn. roots. Int J Chem Sci.

[11] Sharma V, Agrawal RC, Pandey S (2014). Phytochemical screening and determination of anti-bacterial and anti-oxidant potential of *Glycyrrhiza glabra* root extracts. J Envir Res Devel.

[12] Chao H, Wang W, Hou J (2019). Plant growth and soil microbial impacts of enhancing licorice with inoculating dark septate endophytes under drought stress. Front Microbiol.

[13] Li L, Sinkko H, Montonen L (2012). Biogeography of symbiotic and other endophytic bacteria isolated from medicinal *Glycyrrhiza* species in China. FEMS Microbiol Ecol.

[14] Cao XM, Cai J, Li SB (2013). *Fusarium solani* and *Fusarium oxysporum* associated with root rot of *Glycyrrhiza uralensis* in China. Plant Dis.

[15] Parray J, Egamberdieva D, Abd_Allah E (2023). Editorial: Soil microbiome metabolomics: A way forward to sustainable intensification. Front Sustain Food Syst.

[16] Abdullaeva Y, Mardonova G, Eshboev F (2024). Harnessing chickpea bacterial endophytes for improved plant health and fitness. AIMS Microbiol.

[17] Egamberdieva D, Wirth S, Alqarawi AA (2017). Phytohormones and beneficial microbes: Essential components for plants to balance stress and fitness. Front Microbiol.

[18] Rezaei-Chiyaneh E, Mahdavikia H, Subramanian S (2021). Co-inoculation of phosphate-solubilizing bacteria and mycorrhizal fungi: Effect on seed yield, physiological variables, and fixed oil and essential oil productivity of ajowan (*Carum copticum* L.) under water deficit. J Soil Sci Plant Nutr.

[19] Pawlik M, Cania B, Thijs S (2017). Hydrocarbon degradation potential and plant growth-promoting activity of culturable endophytic bacteria of *Lotus corniculatus* and *Oenothera biennis* from a long-term polluted site. Environ Sci Pollut Res.

[20] Chamkhi I, Sbabou L, Aurag J (2023). Improved growth and quality of saffron (*Crocus sativus* L.) in the field conditions through inoculation with selected native plant growth-promoting rhizobacteria (PGPR). Ind Crops Prod.

[21] Egamberdieva D, Shurigin V, Alaylar B (2020). Bacterial endophytes from horseradish (*Armoracia rusticana* G. Gaertn., B. Mey. & Scherb.) with antimicrobial efficacy against pathogens. Plant Soil Environ.

[22] Egamberdieva D, Shurigin V, Alaylar B (2020). The effect of biochars and endophytic bacteria on growth and root rot disease incidence of Fusarium infested narrow-leafed lupin (*Lupinus angustifolius* L.). Microorganisms.

[23] Egamberdieva D, Wirth S, Behrendt U (2017). Antimicrobial activity of medicinal plants correlates with the proportion of antagonistic endophytes. Front Microbiol.

[24] Nejatzadeh-Barandozi F (2013). Antibacterial activities and antioxidant capacity of *Aloe vera*. Bioorganic Med Chem Lett.

[25] Bafana A, Lohiya R (2013). Diversity and metabolic potential of culturable root-associated bacteria from *Origanum vulgare* in sub-Himalayan region. World J Microbiol Biotechnol.

[26] Phetcharat P, Duangpaeng A (2012). Screening of endophytic bacteria from organic rice tissue for indole acetic acid production. Procedia Eng.

[27] Katoch M, Pull S (2017). Endophytic fungi associated with Monarda citriodora, an aromatic and medicinal plant and their biocontrol potential. Pharm Biol.

[28] Farhaoui A, El Alami N, Khadiri M (2023). Biological control of diseases caused by *Rhizoctonia solani* AG-2-2 in sugar beet (*Beta vulgaris* L.) using plant growth-promoting rhizobacteria (PGPR). Physiol Mol Plant Pathology.

[29] Rustamova N, Wubulikasimu A, Mukhamedov N (2020). Endophytic bacteria associated with medicinal plant *Baccharoides anthelmintica* diversity and characterization. Curr Microbiol.

[30] Mora-Ruiz MDR, Font-Verdera F, Díaz-Gil C (2015). Moderate halophilic bacteria colonizing the phylloplane of halophytes of the subfamily *Salicornioideae* (*Amaranthaceae*). Syst Appl Microbiol.

[31] Dashti AA, Jadaon MM, Abdulsamad AM (2009). Heat treatment of bacteria: A simple method of DNA extraction for molecular techniques. Kuwait Med J.

[32] Lane DJ (1991). 16S/23S rRNA Sequencing. Nucleic Acid Techniques in Bacterial Systematic.

[33] Jinneman KC, Wetherington JH, Adams AM (1996). Differentiation of *Cyclospora* sp. and *Eimeria* spp. by using the polymerase chain reaction amplification products and restriction fragment length polymorphisms. Food and Drug Administration Laboratory Information Bulletin LIB no 4044.

[34] Tamura K, Nei M, Kumar S (2004). Prospects for inferring very large phylogenies by using the neighbor-joining method. Proc Natl Acad Sci USA.

[35] Kumar S, Stecher G, Li M (2018). MEGA X: Molecular evolutionary genetics analysis across computing platforms. Mol Biol Evol.

[36] Castric PA (1975). Hydrogen cyanide, a secondary metabolite of *Pseudomonas aeruginosa*. Can J Microbiol.

[37] Schwyn B, Neilands JB (1987). Universal chemical assay for the detection and determination of siderophores. Anal Biochem.

[38] Brown MRW, Foster JHS (1970). A simple diagnostic milk medium for *Pseudomonas aeruginosa*. J Clin Pathol.

[39] Walsh GA, Murphy RA, Killeen GF (1995). Technical note: Detection and quantification of supplemental fungal b-glucanase activity in animal feed. J Anim Sci.

[40] Malleswari D, Bagyanarayan G (2017). *In vitro* screening of rhizobacteria isolated from the rhizosphere of medicinal and aromatic plants for multiple plant growth promoting activities. J Microbiol Biotechnol Res.

[41] Howe TG, Ward JM (1976). The utilization of tween 80 as carbon source by *Pseudomonas*. J Gen Microbiol.

[42] Bano N, Musarrat J (2003). Characterization of a new *Pseudomonas aeruginosa* strain NJ-15 as a potential biocontrol agent. Curr Microbiol.

[43] Egamberdieva D, Kucharova Z (2009). Selection for root colonising bacteria stimulating wheat growth in saline soils. Biol Fertil Soils.

[44] Egamberdieva D, Wirth SJ, Shurigin VV (2017). Endophytic bacteria improve plant growth, symbiotic performance of chickpea (*Cicer arietinum* L.) and induce suppression of root rot caused by *Fusarium solani* under salt stress. Front Microbiol.

[45] Simons M, van der Bij AJ, Brand I (1996). Gnotobiotic system for studying rhizosphere colonization by plant growth-promoting *Pseudomonas* bacteria. Mol Plant Microbe Interact.

[46] Makuwa SC, Motadi LR, Choene M (2023). *Bacillus dicomae* sp. nov., a new member of the *Bacillus cereus* group isolated from medicinal plant *Dicoma anomala*. Int J Syst Evol Microbiol.

[47] Khan MS, Gao J, Chen X (2020). Isolation and characterization of plant growth-promoting endophytic bacteria *Paenibacillus polymyxa* SK1 from *Lilium lancifolium*. Biomed Res Int.

[48] Anandan K, Vittal RR (2019). Endophytic *Paenibacillus amylolyticus* KMCLE06 extracted dipicolinic acid as antibacterial agent derived via dipicolinic acid synthetase gene. Curr Microbiol.

[49] Shurigin V, Alimov J, Davranov K (2022). The diversity of bacterial endophytes from *Iris pseudacorus* L. and their plant beneficial traits. Curr Res Microb Sci.

[50] Egamberdieva D, Kamilova F, Validov S (2008). High incidence of plant growth-stimulating bacteria associated with the rhizosphere of wheat grown in salinated soil in Uzbekistan. Environ Microbiol.

[51] Lim JA, Lee DH, Heu S (2014). The interaction of human enteric pathogens with plants. Plant Pathol J.

[52] Cho ST, Chang HH, Egamberdieva D (2015). Genome analysis of Pseudomonas fluorescens PCL1751: A rhizobacterium that controls root diseases and alleviates salt stress for its plant host. PLoS ONE.

[53] Koberl M, Ramadan EM, Adam M (2013). Bacillus and Streptomyces were selected as broad-spectrum antagonists against soilborne pathogens from arid areas in Egypt. FEMS Microbiol Lett.

[54] Egamberdiyeva D, Hoflich G (2004). Effect of plant growth-promoting bacteria on growth and nutrient uptake of cotton and pea in a semi-arid region of Uzbekistan. J Arid Environ.

[55] Medison RG, Tan L, Medison MB (2022). Use of beneficial bacterial endophytes: A practical strategy to achieve sustainable agriculture. AIMS Microbiol.

[56] Ali S, Duan J, Charles TC (2014). A bioinformatics approach to the determination of genes involved in endophytic behavior in *Burkholderia* spp. J Theor Biol.

[57] Goryluk A, Rekosz-Burlaga H, Blaszczyk M (2009). Isolation and characterization of bacterial endophytes of *Chelidonium majus* L. Pol J Microbiol.

[58] Akinsanya MA, Goh JK, Lim SP (2015). Metagenomics study of endophytic bacteria in *Aloe vera* using next-generation technology. Genom Data.

[59] Liu Y, Mohamad OAA, Salam N (2019). Diversity, community distribution and growth promotion activities of endophytes associated with halophyte *Lycium ruthenicum* Murr. 3 Biotech.

[60] Rana KL, Kour D, Yadav AH (2019). Endophytic microbiomes: Biodiversity, ecological significance and biotechnological applications. Res J Biotechnol.

[61] Michelsen CF, Stougaard P (2012). Hydrogen cyanide synthesis and antifungal activity of the biocontrol strain *Pseudomonas fluorescens* In5 from Greenland is highly dependent on growth medium. Can J Microbiol.

[62] Wozniak M, Gałazka A, Tyskiewicz R (2019). Endophytic bacteria potentially promote plant growth by synthesizing different metabolites and their phenotypic/physiological profiles in the Biolog GEN III MicroPlateTM Test. Int J Mol Sci.

[63] Musa Z, Ma J, Egamberdieva D (2020). Diversity and antimicrobial potential of cultivable endophytic actinobacteria associated with medicinal plant *Thymus roseus*. Front Microbiol.

[64] Glick BR (2014). Bacteria with ACC deaminase can promote plant growth and help to feed the world. Microbiol Res.

[65] Yadav A, Yadav K (2019). Plant growth-promoting endophytic bacteria and their potential to improve agricultural crop yields. Microbial Interventions in Agriculture and Environment.

[66] Fouda A, Eid AM, Elsaied A (2021). Plant growth promoting endophytic bacterial community inhabiting the leaves of *Pulicaria incisa* (Lam.) DC inherent to arid regions. Plants.

[67] Shurigin V, Li L, Alaylar B (2024). Plant beneficial traits of endophytic bacteria associated with fennel (*Foeniculum vulgare* Mill.). AIMS Microbiol.

[68] Sudarshna, Sharma N (2024). Endophytic bacteria associated with critically endangered medicinal plant *Trillium govanianum* (Wall ex. Royle) and their potential in soil nutrition alleviation. Plant Stress.

[69] Deepa N, Chauhan Sh, Singh A (2024). Unraveling the functional characteristics of endophytic bacterial diversity for plant growth promotion and enhanced secondary metabolite production in *Pelargonium graveolens*. Microbiol Res.

